# Probing Early Misfolding Events in Prion Protein Mutants by NMR Spectroscopy

**DOI:** 10.3390/molecules18089451

**Published:** 2013-08-07

**Authors:** Gabriele Giachin, Ivana Biljan, Gregor Ilc, Janez Plavec, Giuseppe Legname

**Affiliations:** 1 Department of Neuroscience, Scuola Internazionale Superiore di Studi Avanzati (SISSA), Via Bonomea 265,Trieste I-34136, Italy; E-Mail: giachin@sissa.it; 2 Department of Chemistry, Faculty of Science, University of Zagreb, Horvatovac 102A, Zagreb HR-10000, Croatia; E-Mail: ibiljan@chem.pmf.hr; 3 Slovenian NMR Centre, National Institute of Chemistry, Hajdrihova 19, Ljubljana SI-1000, Slovenia; E-Mails: gregor.ilc@ki.si (G.I.); janez.plavec@ki.si (J.P.); 4 EN-FIST Center of Excellence, Ljubljana SI-1000, Slovenia; 5 Faculty of Chemistry and Chemical Technology, University of Ljubljana, Ljubljana SI-1000, Slovenia

**Keywords:** prion protein, prions, genetic mutations, polymorphisms, prion diseases, 3D structure, NMR spectroscopy

## Abstract

The post-translational conversion of the ubiquitously expressed cellular form of the prion protein, PrP^C^, into its misfolded and pathogenic isoform, known as prion or PrP^Sc^, plays a key role in prion diseases. These maladies are denoted transmissible spongiform encephalopathies (TSEs) and affect both humans and animals. A prerequisite for understanding TSEs is unraveling the molecular mechanism leading to the conversion process whereby most α-helical motifs are replaced by β-sheet secondary structures. Importantly, most point mutations linked to inherited prion diseases are clustered in the C-terminal domain region of PrP^C^ and cause spontaneous conversion to PrP^Sc^. Structural studies with PrP variants promise new clues regarding the proposed conversion mechanism and may help identify “hot spots” in PrP^C^ involved in the pathogenic conversion. These investigations may also shed light on the early structural rearrangements occurring in some PrP^C^ epitopes thought to be involved in modulating prion susceptibility. Here we present a detailed overview of our solution-state NMR studies on human prion protein carrying different pathological point mutations and the implications that such findings may have for the future of prion research.

## 1. Introduction

The physiological cellular prion protein (PrP), PrP^C^, is a glycosylphosphatidylinositol (GPI)-anchored glycoprotein localized on the outer leaflet of the cellular membrane in mammalian cells. The mature human (Hu) PrP^C^ (HuPrP) is composed of 209 residues including a largely unstructured N-terminal part and a globular α-helix rich C-terminal domain. Despite being highly conserved among mammals, its physiological function has not been established with certainty. Defining PrP^C^ functions remains an absolute requirement for understanding transmissible spongiform encephalopathies (TSEs), or prion diseases, which are caused by the posttranslational conversion of PrP^C^ into a misfolded and pathogenic isoform denoted prion or PrP^Sc^.

The term “prion” was coined by Stanley B. Prusiner in 1982, when he introduced the heretical notion of a small proteinaceous infectious particle as sole causal agent of TSEs in human and animals [1]. In humans, TSEs include a heterogeneous group of invariably fatal neurodegenerative diseases etiologically arising as sporadic, genetic or acquired. According to the recent classification, idiopathic forms include sporadic Creutzfeldt-Jakob disease (sCJD), sporadic fatal insomnia (sFI) and the variably protease sensitive prionopathies (VPSPr). Genetic forms comprise familial CJD (fCJD), Gerstmann-Sträussler-Scheinker disease (GSS), fatal familial insomnia (FFI) and prion protein cerebral amyloid angiopathy (PrP-CAA). The acquired forms are transmitted from human to human, as iatrogenic CJD (iCJD) or Kuru, from cattle to human, and human to human, as variant CJD (vCJD) [2]. In animals, relevant TSEs are scrapie in sheep and goats, bovine spongiform encephalopathy (BSE) in cattle, and chronic wasting disease (CWD) in deer, elk and moose [3].

From the epidemiological point of view, human TSEs are rare diseases evenly distributed worldwide. Sporadic CJD is the most frequent form and accounts for an incidence of about 0.6–1.2 per million per year [4]. Genetic forms account for 10%–15% of human prion diseases, whereas the acquired forms are negligible [5].

The main focus of research in prion biology is understanding the molecular mechanisms involved in the conformational conversion of PrP^C^ to its pathological counterpart, PrP^Sc^, and how prions trigger neurotoxic signals leading to TSEs. Genetic forms of human prion diseases are linked to specific mutations in the PrP gene (*PRNP*). Key evidence exists supporting the link between mutations and spontaneous conversion of PrP^C^ to PrP^Sc^ in the brain. Over the past few years, biological studies on PrP pathological mutants have emerged as an invaluable tool for the comprehension of the molecular basis of TSEs. By means of different experimental approaches, several groups explored the use of the mutations in order to understand their effects on PrP structure and stability, elucidate the cellular events leading to PrP accumulation and misfolding, and explain the PrP^C^ physiological roles and its neurotoxic potential [6,7,8,9,10].

In the next sections, we present an overview of our recent NMR structural studies on HuPrP pathological mutants. We solved and analyzed the structures corresponding to the globular domain of different HuPrP constructs including the wild-type (WT), the V210I and Q212P mutants (linked to fCJD and GSS, respectively) and the E219K polymorphism. As a prelude, we first review what is currently known on PrP^C^ structure and functions, PrP^Sc^ structural models, as well as genetic prion diseases.

## 2. Structural Features of PrP^C^

*PRNP* has been mapped in the short arm of chromosome 20 and the protein coding frame is present within a single exon [11]. PrP^C^ is highly expressed within the central and peripheral nervous systems, although its content varies among distinct cell types, neurons and brain regions [12]. Expression of the protein is developmentally regulated in different mammalian species [13,14,15,16,17]. The immature form of PrP^C^ is composed of 253 residues including an N-terminal endoplasmic reticulum (ER) signal peptide and a C-terminal GPI-anchoring signal peptide, which are cleaved during protein maturation and translocation to the extracellular side of the plasma membrane [18]. Similar to other GPI-anchored proteins, PrP^C^ is found mainly attached to low density detergent insoluble membrane domains (DRMs) or lipid rafts [19].

**Figure 1:**
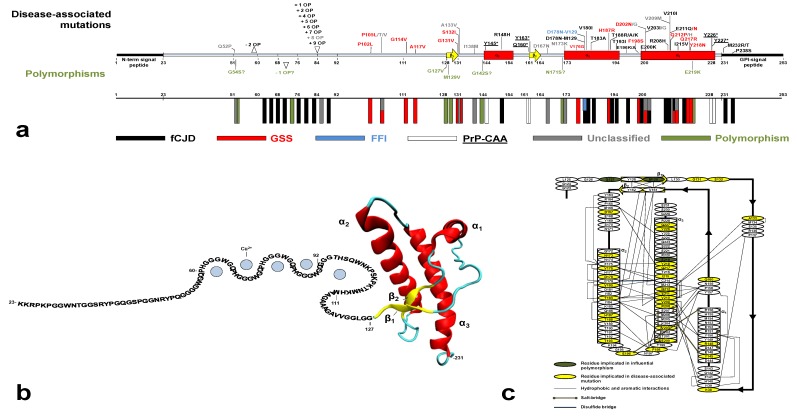
(**a**) Secondary structure of the immature HuPrP with all the currently identified disease-associated mutations and polymorphisms (unclassified phenotype in gray; fCJD in black; GSS in red; PrP-CAA underlined; FFI in blue; and polymorphisms in green). The mature HuPrP consists of residues 23–231, while the N-terminal and C-terminal signal peptides are cleaved during protein maturation. In the lower panel, the frequency of the mutations and polymorphisms along the *PRNP* sequence is represented by colored rectangles. (**b**) Cartoon of the full-length HuPrP with the sequence-based representation of the disordered N-terminus and the structured C-terminal domain from PDB code 2LSB [28]. Up to four Cu^2+^ ions are bound to the OR (residues 60–92) and one Cu^2+^ ions is bound to the non-OR (residues 93–111). (**c**) Schematic representation of the secondary elements in the C-terminal HuPrP structured domain. The stabilizing long-range interactions were inferred from structural analysis and published data [31,32,33]. Residues involved in disease-influential mutations or polymorphisms are labeled.

Most structural information on PrP^C^ came from bacterially expressed recombinant (rec) PrP. Despite the lack of Asn-linked glycosylation at residues 181 and 197, recPrP is structurally equivalent to brain-derived PrP^C^ [20]. Atomic structures obtained by NMR techniques and X-ray crystallography revealed that recPrP shares a very similar fold across different mammalian species [7]. The full-length PrP^C^ has a unique structure: while the N-terminal moiety is largely unfolded from residue 23 to 127, the C-terminus possesses a well-defined secondary and tertiary structure (residues 128–231, Figure 1b).

The N-terminus features the octapeptide-repeat region (OR), an evolutionarily conserved motif whose repeated units can vary among species [21]. In HuPrP, the OR consists of one nonapeptide, PQGGGGWGQ (residues 51–59) and four repeats of sequence PHGGGWGQ (residues 60–92). Within the four repeats, histidines and tryptophan residues were found to be essential for the high affinity binding of copper ions and, to a lesser extent, of other divalent cations [22,23]. Additional histidines at position 96 and 111 are involved in high affinity copper binding and form the non-OR, or fifth, copper binding site [24,25] (Figure 1b). The positively charged segment, spanning residues 96–111, precedes a hydrophobic sequence (residues 112–127). These two biochemically distinct segments act in concert to control the co-translational translocation at the ER during PrP^C^ biosynthesis [26,27].

The HuPrP C-terminal domain is composed of three α-helices (α_1_, α_2_ and α_3_) and two very short β-strands which form an antiparallel β-sheet (β_1_ and β_2_). Helices α_2_ and α_3_ form the bulk of the globular domain and are covalently bridged by a disulfide bond between Cys179 and Cys214. The C-terminus NMR structures of WT HuPrP obtained at different pH values (4.5, 5.5 and 7) show almost identical backbone tertiary structures with local differences in flexible regions [28,29,30]. 

A detailed analysis reveals that HuPrP core structure is stabilized by extensive hydrophobic, aromatic and salt bridges networks between the β_2_-α_2_-α_3_ secondary elements and α_3_-α_1_ helices. These stabilizing interactions govern the proper folding of the C-terminal domain. Importantly, disease-related mutations are clustered almost in the folded part and may contribute to destabilize the native HuPrP conformation (Figure 1c) [31,32,33,34].

Among different species, local sequence and structural variations are prominently localized at the interface between the β_2_-α_2_ loop (residue 163–171) and the C-terminal part of α_3_-helix (residues 218–231), providing insights into a region of potential importance for pathogenicity and barriers to TSE transmission across species. Although this loop is highly flexible in most species, it shows a well-defined conformation in the recPrP of Syrian hamster [35], elk [36], bank vole [37], wallaby [38], rabbit [39] and horse [40]. In elk and bank vole recPrP, this rigidity was shown to be controlled by a single aminoacid substitution (S170N), whereas in wallaby, rabbit and horse recPrP it correlates with long-range interactions between residue 166 and 225. Importantly, no occurrence of TSE has been reported in rabbit, horse or any marsupial species, suggesting that prion resistance is enciphered by the amino acid composition of the β_2_-α_2_ loop and its stabilizing contacts with the C-terminal segment of α_3_-helix [41].

The PrP^C^ N- and C-terminal domains are often considered structurally independent and non-interacting in prions. This view is supported by a body of evidence showing that the structured domain plays a crucial role during the pathological conversion to prions. In fact, limited protease K (PK) digestion of PrP^Sc^ often produces a smaller, protease-resistant segment of approximately 142 amino acids spanning residue ~90 to 231 and referred to as PrP 27–30. This molecule is sufficient to generate infectious amyloid fibrils [42,43]. Additionally, *in vitro* generated amyloid fibrils composed only of C-terminal rec murine (Mo) PrP (residues 89–230) were pathogenic and infectious in transgenic (Tg) mice Tg9949 overexpressing MoPrP(89–230) [44]. Although the N-terminal region is not essential for prion conversion and infectivity, in the self-association of PrP^C^ to PrP^Sc^ it may act as a modulator of both conversion and prion propagation [45].

Recent studies are attributing a significant role to the N-terminus in driving tertiary contacts with the C-terminus. By means of synchrotron-based X-ray absorption fine structure (XAFS) techniques, we showed that Q212P mutation, which is localized in α_3_-helix, alters the proper copper coordination in the non-OR copper binding site. These findings imply a structural effect of C-terminal mutations on the N-terminal part where functional domains are present [24]. Copper was shown to promote the association of the N-terminus to α_1_-helix [46]. Finally, zinc binding to the OR region induces novel electrostatic-driven contacts between this domain and the α_2_-α_3_ helices [47].

## 3. Defining PrP^C^ Functions

The physiological role of PrP^C^ is usually disregarded at the expense of its role in TSEs. However its definition is essential to understand the molecular mechanisms leading to the disease. Common strategies often employed to identify PrP^C^ functions include the development of different Tg mice lines which are knockout (KO) for PrP gene (*Prnp*). Despite the wide distribution of PrP^C^ in the mammalian central nervous system (CNS), *Prnp* KO mice (*Prnp*^0/0^) surprisingly display no major anatomical developmental deficits [48]. Further evaluations revealed that *Prnp*^0/0^ mice show mild behavioral phenotypes such as deficits in spatial learning and circadian rhythms [49], altered long-term potentiation [50,51] and increased excitability of hippocampal neurons [52,53,54,55]. These studies highlight the synaptic role of PrP^C^ in the development of the hippocampus and are corroborated by immunohistochemical studies on PrP^C^ expression and localization in the hippocampus during the development [13,14,15,16,17].

A neuroprotective PrP^C^ function has been inferred from peculiar features of *Prnp*^0/0^ mice such as increased neuronal damage after ischemic stroke [56,57], increased mortality in excitotoxic conditions [58], enhanced susceptibility to kainate-induced seizure [59,60] and to glutamate treatment [61] and altered *N*-methyl-d-aspartate (NMDA) receptor currents with greater basal excitability, increased amplitude and slowed kinetics [62]. The observed neuroprotective roles of PrP^C^ are consistent with the currently accepted idea that PrP^C^ is a copper binding protein involved in copper homeostasis. A wealth of studies explored the functional aspects of this interaction indicating that the protein acts as a copper transporter, a sink of copper excess or a scavenger for copper-generated free radicals [63].

Insights into a possible PrP^C^ involvement in cell proliferation and differentiation derive from results on embryonic hippocampal neuron cultures treated with recPrP showing an induced polarization in synapse development and neuritogenesis [64].

Finally, putative PrP^C^ functions are based on its numerous interacting protein molecules [65,66]. Being a GPI-anchored protein present in the DRM, PrP^C^ interacts both *in cis* or *in trans* with a variety of signaling molecules including, for instance, cavelolin-1, Fyn and Src tyrosines kinases [67], neuronal cell adhesion molecules (NCAMs) [68,69], stress-inducible protein 1 [70] and, recently, reelin [71], the NR2D subunit of the NMDA receptor [62] and the α_2_δ-1 subunit of the voltage gated calcium channel [72]. Taken together these studies reinforce the idea that PrP^C^ is a pleiotropic protein with different functions, likely acting as a dynamic cell surface scaffolding protein for the assembly of signaling modules.

## 4. Prion Conversion and PrP^Sc^ Molecular Models

PrP^C^ is the first biological system where a polypeptide exists in at least two distinct conformations associated with either physiological functions or disease. The central molecular event in the replication of mammalian prions is the self-propagating conformational conversion of PrP^C^ to the misfolded PrP^Sc^ form. This postulate is known as the “protein-only” hypothesis [1].

Two different mechanisms of prion replication have been put forward. The “template assistance model” proposes that PrP^Sc^ exists as a monomer that is thermodynamically more stable than PrP^C^, but this favored conformer is kinetically inaccessible. In the rare event that a PrP^Sc^ is formed spontaneously (or provided exogenously) it can template the misfolding of PrP^C^ by direct interaction. In this model, the critical step in the conversion is the formation of a dimer between PrP^Sc^ and PrP^C^, or a partially destabilized folding intermediate of PrP^C^ denoted as PrP^*^. PrP^Sc^ acts as a template that catalyzes the refolding of PrP^C^ to a thermodynamically more stable PrP^Sc^ conformation [73].

In the “nucleation-polymerization model” the conversion between PrP^C^ and PrP^Sc^ is reversible, but the PrP^Sc^ monomer is much less stable than PrP^C^ (*i.e.*, the equilibrium is strongly toward PrP^C^). Stabilization of PrP^Sc^ occurs only upon formation of a stable oligomeric nucleus, which is thermodynamically not favorable. However, once the nucleus has formed, additional monomeric PrP^C^ is recruited and adopts the conformation of PrP^Sc^. The rate-limiting step in this mechanism is not the conformational conversion itself but the nucleation step. This step, responsible for the lag phase in the spontaneous conversion, can be bypassed and accelerated by adding preformed PrP^Sc^ seeds [74].

One of the most important challenges in prion biology is determining the structural traits of infectious prions. Detailed information about PrP^Sc^ structure is crucial not only for understanding the molecular basis of conversion and transmission, but also for potential pharmacological intervention. Several spectroscopic studies revealed that the amyloid form of PrP^Sc^ is β-sheet enriched [75,76,77,78,79,80]. Numerous research groups investigated the molecular mechanism of the conversion by different methods (reviewed in [8]). However, all the structural studies on prions are limited by the disorder and insolubility of PrP^Sc^ and they still failed to identify its structure at the atomic level. Due to the lack of atomistic details for PrP^Sc^, different prion models have been proposed based on low-resolution experimental approaches [81]. One such model is denoted as β-helical model. It is based on fiber X-ray diffraction and imaging simulation techniques on brain-extracted Syrian hamster fibrils. It proposes that the segment ~90–175 forms a four-stranded β-sheet core organized in a β-helical configuration, whereas α_2_ and α_3_ helices retain their native folding [82]. By contrast, hydrogen deuterium exchange experiments on brain-derived GPI-anchorless PrP^Sc^ showed that the region from residue ~90 to the entire C-terminus displays slow exchange rates which are typical for a structure consisting of a continuum of β-strands [83]. While recent X-ray diffraction data on different prion strains (Wille, H. University of Alberta, Edmonton, Alberta, Canada, unpublished data) seem to confirm the presence of cross-β structure consistent with the β-helical model, the latest work by Surewicz’s group [83] indicates that other structural PrP^Sc^ models cannot be ruled out.

## 5. Genetic Human Prion Diseases: An Overview

Genetic forms of HuTSEs are transmitted as autosomal dominant traits and co-segregate with missense or insertional/deletional mutations in *PRNP*. Over the last decades a number of CJD surveillance reports have identified up to 58 mutations linked to human prion diseases, associated with a heterogeneous spectrum of clinical and pathological phenotypes (Figure 1a). Missense mutations include 44 non-synonymous codon substitutions and five non-sense (or “stop”) mutations. Insertional or deletional mutations are exclusively localized in the OR, which normally contains one nonapeptide and four octapeptides. The insertions identified so far include one, two and four to nine extra octapeptides (OP), while only a deletion of two OP was reported [84]. Pathological point mutations have been found along the entire *PRNP* sequence but are mainly clustered in the C-terminal structured domain (residues 128 to 231) where about 39% of residues are implicated in disease-associated mutations. Interestingly, almost all the mutations that have been identified so far in the globular domain affect the hydrophobic and aromatic interactions and the salt bridges, which act in concert to stabilize the proper α-helical PrP^C^ fold (Figure 1c).

Pathological point mutations are classified into five clinical categories including fCJD, GSS, FFI, PrP-CAA and unclassified phenotype (Figure 1a). Although the mutations seem defined according to a rigid clinical classification, very often the syndromes overlap considerably. Only for a few mutations the prion transmissibility to model animals has been proved [2], therefore many genetic human prion diseases should be considered as proteinopathies. In general, these genetic forms show incomplete or age-dependent penetrance, as frequently family members carrying a *PRNP* mutation do not develop prion diseases [85].

### 5.1. Clinico-Pathological Features of Genetic Human Prion Diseases

The core features of fCJD include pathologically spongiform neurodegeneration, astrogliosis, neuronal loss and amyloid plaques, and clinically progressive dementia with visual, cerebellar and extrapyramidal signs and myoclonus [86]. Phenotypic diversity within sCJD and fCJD has been observed and most fCJD forms share features with specific subtypes of sCJD [87]. Several fCJD-associated mutations have been described. The most common fCJD-related mutations are E200K, V210I, E196K and +6OP insertion, which show distinct geographic clusters with high incidence of the disease [5,88]. Disease duration is highly variable—from weeks to less than two years [86].

FFI is caused by D178N mutation in combination with methionine at codon 129. It is characterized by insomnia, dysautonomia and motor signs associated mainly with thalamic astrogliosis, atrophy and spongiosis [89]. The established haplotypic relationships between D178N-M129 segregating with FFI and D178N-V129 segregating with fCJD [90] have been questioned by patients segregating with both FFI and fCJD phenotypes [91] or with different *PRNP* mutations showing a FFI-like syndrome [92]. Disease duration ranges from six to 42 months [86].

The histopathological presence of PrP^Sc^ plaque deposits, rather than the heterogeneous clinical features, defines GSS. Several point mutations are associated with this syndrome. The archetypal and most frequent GSS-related mutation is P102L. Other less frequently reported point mutations are P105L, A117V, H187R and F198S, while other mutations have been described in very few family cases [86,93].

All five non-sense mutations (Y145*, Q160*, Y163*, Y225* and Y226*) have attracted particular interest as they are associated with PrP^Sc^ amyloid deposition in the walls of arteries, arterioles, veins and capillaries of the CNS [93,94]. The vascular amyloid fibril deposits have been described in other neurodegenerative diseases, including Alzheimer’s disease, and are generally defined as cerebral amyloid angiopathy (CAA) or, in the case of PrP carrying premature stop codons, PrP-CAA [93]. Interestingly, a patient affected by Alzheimer’s disease with CAA showed PrP^Sc^ co-localization in the same amyloid deposits [95]. The reasons of PrP^Sc^ perivascular distribution are still unknown, although different Tg mice expressing anchorless PrP are now available [96,97,98,99]. The unique disease phenotype observed in PrP-CAA clearly indicates that the lack of GPI-moiety greatly contributes to the formation of PrP^Sc^ amyloid outside the cells and provides an intriguing link between prion diseases and other neurodegenerative diseases.

For several other disease-associated mutations the clinico-pathological classification is still missing, because published data are insufficient and they are based only on genetic tests without detailed neuropathological and biochemical analyses. Often the new *PRNP* mutations have been reported in single patients without any family history for prion diseases, and are not associated with the presence of detectable PrP^Sc^ in the brain. Therefore the pathological role of such rare mutations remains to be assessed. Recently new unclassified mutations have been discovered including Q52P in the OP, D167N in the β_2_-α_2_ loop, N173K in α_2_-helix, V203G, V209M and Q212H in α_3_-helix [100,101]. Other confounding aspects are multiple mutations affecting the same codon and segregating with completely different clinico-pathological features, for instance P105L (linked to GSS) and P105T/V (unclassified disease phenotype), D202N (GSS) and D202G (unclassified), E211Q (CJD) and E211N (GSS), Q212P (GSS) and Q212H (unclassified).

### 5.2. Influential Polymorphisms in PRNP

While point mutations are responsible for genetic prion diseases, some polymorphisms in *PRNP* influence the etiology and neuropathology of the disease. The polymorphic residue at codon 129 (M129V) has been studied extensively. It has been shown that M129V may affect the susceptibility to prion diseases [102] and the incubation time of vCJD [103,104]. Methionine homozygosis at codon 129 is considered a risk factor for sCJD and iCJD [85,100,105].

Another naturally occurring HuPrP polymorphism, E219K, was found to protect against prion diseases. Heterozygosis at codon 219 was initially reported in the general Japanese population (allele frequency ~6%) [106,107] and was later found also in other Asiatic populations [108,109], but very rarely in Western Europeans [100,105]. The absence of confirmed cases of sCJD patients carrying the E219K polymorphism in Japan or Korea suggests that E219K heterozygosis acts as a protective factor to sCJD [110]. This polymorphism influences also the clinico-pathological features of both genetic [111,112,113] and acquired [114,115,116,117] prion diseases. Polymorphism at codon 127 (G127V) has been reported only among the natives of Papua New Guinea and is protective against Kuru [118]. Uncommon polymorphisms include the absence of 1OP [119], G54S, N171S [100] and G142S [108] which seem to have no susceptibility or unknown effects in HuTSEs.

## 6. Structural Biology of HuPrP Pathological Mutants

In the previous sections we overviewed the current knowledge on mutations and polymorphisms in *PRNP* and their clinico-pathological implications. Our understanding of the molecular mechanisms whereby mutations cause genetic TSEs still remains limited. It has been proposed that *in vitro* mutations may increase the likelihood of misfolding by thermodynamic destabilization of HuPrP. While this is true for few studied mutants [120,121,122], mutations may also alter the protein surface properties, triggering in turn abnormal interactions with other not yet identified cofactors [123,124] or causing aberrant cellular trafficking and accumulation inside the cells [125].

**Figure 2:**
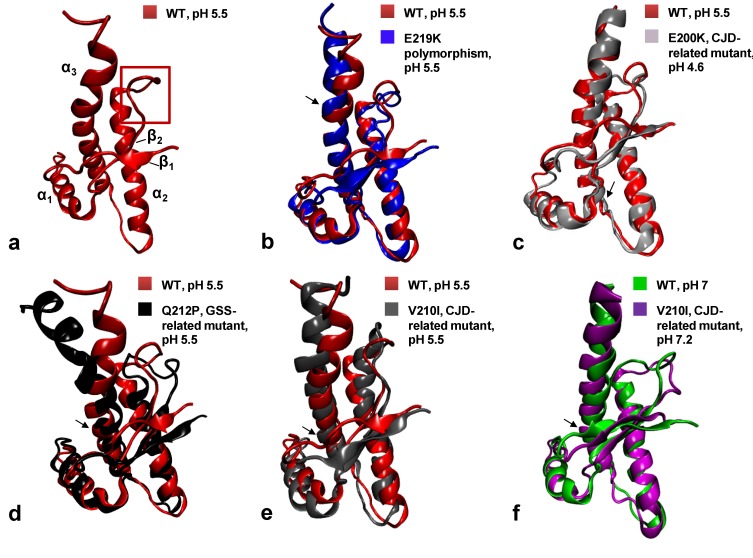
Cartoon representations and backbone heavy atom overlays of the different WT and mutants HuPrP folded domains (residues 126–231): WT at pH 5.5 in red (**a**, PDB code 2LSB), E219K in blue (**b**, PDB code 2LFT), E200K in light gray (**c**, PDB code 1FO7), Q212P in black (**d**, PDB code 2KUN), V210I at pH 5.5 in gray (**e**, PDB code 2LEJ) and (**f**) V210I at pH 7.2 in magenta (PDB code 2LV1) superimposed with the WT at pH 7 in green (PDB code 1HJN). The red frame on the WT (**a**) highlights the β_2_-α_2_ loop region. Black arrows point at the location of each mutation.

Structural studies on HuPrP carrying pathological mutations or polymorphisms may help to identify the regions involved in the misfolding to PrP^Sc^. Recently, we attempted to determine the high resolution 3D NMR structure of the HuPrP carrying the Q212P and V210I mutations [126,127,128] linked to GSS and fCJD, respectively. Additionally, we solved the NMR structure of the CJD-protective polymorphism E219K and the WT HuPrP obtained under the same experimental conditions as the mutants [28]. Before our NMR structures, there was no clear evidence that a pathological point mutation may cause substantial structural difference in the HuPrP fold. Indeed, the fCJD-related E200K mutant (Figure 2c) exhibits minimal differences in the NMR structure [129] and dynamics to the WT protein [130]. The comparison of mutant structures with the WT HuPrP revealed that mutations introduce novel local structural differences, although the global tertiary fold remains very similar (Figure 2).

All the recHuPrP we analyzed were bacterially expressed in isotopic medium, purified from inclusion bodies and *in vitro* refolded [127,128]. Proteins always adopted a globular fold as indicated by the amide signal dispersion in the ^1^H-^15^N HSQC spectrum, providing a preliminary indication of sample suitability for NMR experiments. The backbone assignment was achieved with standard triple resonance experiments, including HNCA, HN(CO)CA, HNCACB and CBCA(CO)NH NMR experiments. ^1^H and ^13^C resonances of sidechains were assigned by analyses of 3D (H)CCH-TOCSY and ^13^C-edited NOESY-HSQC spectra. The large number of NOE restraints together with the completeness of resonance assignments (>95%) allowed us to determine the structure of all HuPrP mutants, the WT and E219K polymorphism with high resolution.

### 6.1. The Q212P Mutant NMR Structure

The Q212P was the first mutation to attract our interest for structural investigations. Due to its unique sidechain features, proline is a well-known α-helix breaker. Pro212 interrupts the hydrogen (H)-bond with Arg208, increases the helix pitch and induces local rigidity in the α_3_-helix. Therefore we expected major structural rearrangements caused by this mutation, although it is associated with a very rare form of GSS characterized by mild amyloid PrP^Sc^ deposition and reduced penetrance [131,132]. Interestingly, the NMR structure of the Q212P mutant obtained at pH 5.5 revealed only minor changes at local level, between the segment from residue 205 to 220, but notable differences in distal regions. In particular, α_3_-helix showed a broken conformation at residue 221 and 222 which led to the formation of a novel highly flexible α-helix motif from residues 223 to 228 (Figure 2d).

**Figure 3:**
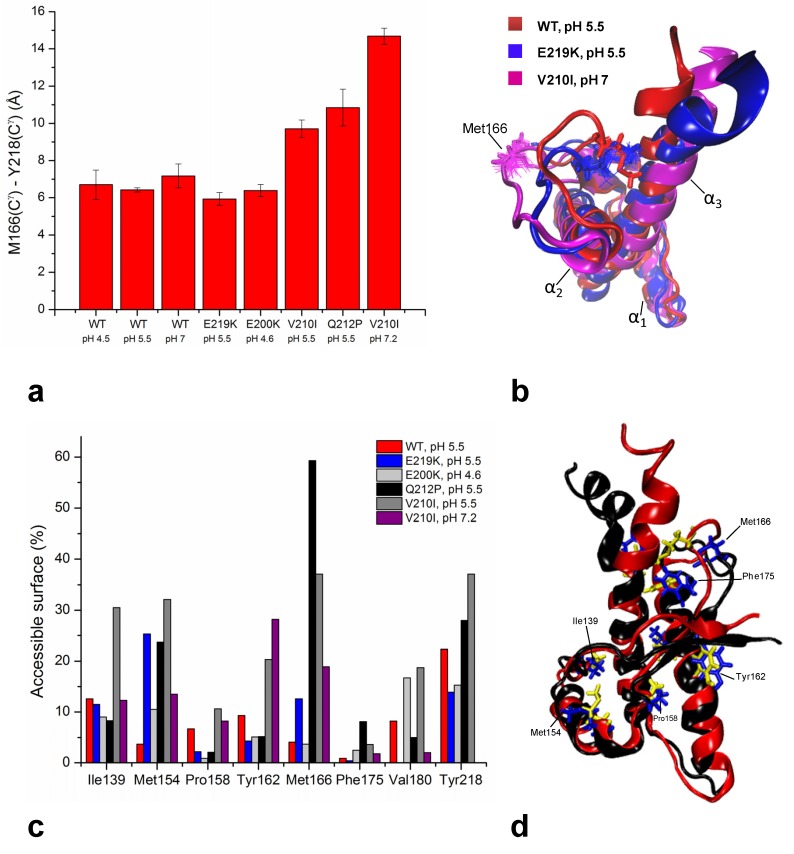
(**a**) Distances in Å between residues Met166 and Tyr218 within the β_2_-α_2_ loop and of α_3_-helix in different WT and mutant HuPrP structures. Distances were calculated using VMD software [138]. (**b**) Cartoon representations and backbone heavy atoms overlays of WT at pH 5.5, E219K and V210I at pH 7.2. This peculiar view highlights the different spacing observed among the HuPrP structures between the β_2_-α_2_ loop and those of α_3_-helix. (**c**) Average values of solvent accessibility (%) for selected residues in different WT and mutant HuPrP structures calculated using GETAREA software [139]. (**d**) Cartoon representations and backbone heavy atom overlays of WT and Q212P structures. Selected hydrophobic and aromatic residues are highlighted.

Upon P212 mutation, α_3_-helix exhibits small rotation along the helical axis with respect to the WT protein. A turn of α_3_-helix around Pro212 is altered to accommodate unfavorable steric interactions of proline with the preceding residue Glu211. The relative orientation of α_3_-helix with respect to the α_2_-helix changed from 51° in the WT to 33° in the Q212P mutant. As a result, several aromatic and hydrophobic interactions at the α_2_-α_3_ interface are lost. Major structural changes are observed in the β_2_-α_2_ loop region where the aromatic and hydrophobic clusters composed by Tyr163, Met166, Phe175, Tyr218 and Tyr225 are changed, showing increased exposure to the solvent and longer distance between the loop and the C-terminal part of α_3_-helix (Figure 3).

### 6.2. NMR Structures of V210I at Two Physiological pH Values

The V210I mutant is linked to fCJD, and it shows high incidence across European countries, e.g., in Italy where geographic clusters carrying this mutation have been identified [133]. Val210 is located in the middle of the α_3_-helix and it is part of tightly packed hydrophobic and aromatic residues present at the interfaces between the α_2_ and α_3_ helices and β_2_ strand (Figure 1c). Therefore we expected this mutation to promote misfolding by altering these stabilizing interactions.

We investigated the effect of V210I on HuPrP structure at two different physiological pH values. Acidic pH conditions are characteristic of the intracellular endosomial compartments [134] where PrP^C^ is sequestered during its internalization. Reports using cell culture models proposed that PrP^C^ misfolding and accumulation may occur during endocytosis in both late and recycling endosomes [135,136,137].

At pH 5.5 the Ile210 causes steric crowding in the inter-helical interface. V210I mutation induces reorientations of several residues involved in α_2_–α_3_ hydrophobic interactions. In order to accommodate unfavorable steric interactions with Ile210, the sidechain of Val180 changed its conformation along C_α_-C_β_ bond by 180°. In addition, the mutation significantly altered the orientation of sidechains of Val176 and Ile184, thus influencing their hydrophobic contacts with other residues. Interestingly, as a consequence of these rearrangements the α_2_-helix is distorted (Figure 2e). Also in this case the inter-helical angle changes from 51° in the WT to 23° in V210I. Another region within the globular domain of V210I mutant that displays interesting structural features is the interface of α_1_ and α_3_ helices. Helix α_1_ in the mutant exhibits a reduced propensity to form tertiary hydrophobic interactions with the protein core. The β_2_–α_2_ loop region in V210I is poorly defined, shows an altered conformation and an increased distance from the α_3_-helix as observed also in the Q212P mutant (Figure 3).

Comparison with V210I obtained at pH 7.2 showed that changing pH from 5.5 to neutral condition barely affects the global architecture of the protein (Figure 2f). However, a careful inspection revealed local structural differences between the two V210I structures. The most prominent pH-related changes involve alterations in interactions within the hydrophobic core. At pH 5.5, several hydrophobic contacts between residues at the α_2_–α_3_ interface are lost. Moreover, at neutral pH the V210I mutant exhibits a more compact packing of hydrophobic residues at the interface of the β_1_–α_1_ loop and α_1_ and α_3_ helices. Additional stabilizing interactions at these interfaces are provided through hydrophobic contacts between Phe141 and Tyr145, Tyr145 and Tyr149, Tyr145 and Met209, and Met154 and Val209, which are not present at acidic condition. In addition to electrostatic interactions that stabilize the highly hydrophilic part of α_1_-helix (Asp147−Arg151 and Glu146-Lys204 salt bridges), hydrophobic interactions are important for maintaining the stability of α_1_-helix and for its interactions with other parts of the globular domain of HuPrP (Figure 1c). Taken together, the resulting comparisons of NMR structures of V210I obtained at two pH values indicate that the separation of the β_1_−α_1_ loop, α_1_-helix, and the α_1_−β_2_ loop from the α_2_−α_3_ region is a hot “spot” during the transition from neutral to acidic pH, and it is required during the early stages of the conversion process. In support of this view, molecular dynamics (MD) experiments highlighted the role of α_1_-helix in the early steps of PrP^C^ misfolding induced by a decrease in pH [140,141,142]. According to these MD studies, lowering the pH induces a loss of contacts between α_1_-helix and other secondary structure elements in the globular domain of HuPrP, as we observed in our V210I mutant structures.

### 6.3. NMR Structures of WT HuPrP and of HuPrP Containing E219K Protective Polymorphism

The naturally occurring HuPrP polymorphism, E219K, attracted interest because it was found to protect against prion diseases. Heterozygosis at codon 219 was initially reported in the Japanese population [107]. The lack of confirmed cases of sCJD patients carrying the E219K polymorphism led to the conclusion that E219K heterozygosis acts as a protective factor against sCJD. This characteristic, also denoted as “dominant-negative effect”, has been reported in several experimental studies both *in vivo* and *in vitro* [123,143,144,145,146,147]. The structural basis responsible for the protective effect of the E219K polymorphism has so far remained elusive. To gain insight into the structural determinants underlying the protective influence of this naturally occurring polymorphism, we determined its high-resolution 3D structure based on NMR datasets. Because we expected minor local structural changes, we solved also the WT HuPrP obtained under exactly the same experimental conditions used for mutants and E219K polymorphism (Figure 2a).

The 3D structure of WT HuPrP at pH 5.5 is very similar to the previously reported structure of the WT protein resolved at pH 4.5 [30]. The two structures are superimposable on the backbone atoms with an r.m.s.d. value of 1.1 Å. The most apparent differences occur within the β_2_-α_2_ loop region referring to a different orientation of the Tyr169. At pH 4.5 this residue points toward the interior of the protein, whereas at pH 5.5 it is exposed to solvent, thus loosing contacts with nearby aromatic residues.

The folded domains of E219K and WT revealed that they are very similar, with backbone r.m.s.d. of only 1.33 Å. Substitution of Glu by Lys at position 219 apparently does not induce substantial local structural changes (Figure 2b). Nevertheless, the 3D structure of E219K pointed to rearrangements of some residues in the β_1_-α_1_ loop (Phe141), α_1_-helix (Tyr145 and Tyr149) and α_1_-β_2_ loop (Tyr157) that affected their mutual hydrophobic interactions with residues in α_3_-helix. NOE data revealed close packing between the sidechains of several residues situated at the interface of β_1_-α_1_ loop, α_1_-helix, α_1_-β_2_ loop and α_3_-helix. The sidechain of Phe141 is directed towards the α_1_-α_3_ inter-helical interface engaged in π-π interactions with Tyr149 and Tyr150. These three aromatic residues form a hydrophobic cluster. Tertiary contacts are also manifested through pronounced hydrophobic interactions of Phe141, Tyr149 and Tyr150 with Met205, and Phe141 and Tyr150 with Val209. Moreover, the interface of β_1_-α_1_ loop, α_1_-helix, α_1_-β_2_ loop and α_3_-helix is further stabilized through hydrophobic interactions of Tyr157 with Met206 and Val209. The structurally poorly defined β_2_-α_2_ loop region also exhibited rearrangements involving both hydrophobic and electrostatic interactions. Especially pronounced contacts involve Met166 and Phe175 located in the loop region and Tyr218 and Tyr225 in the α_3_-helix (Figure 3). Although the E219K did not induce significant local structural changes, this polymorphism has a marked effect on the distribution of electrostatic surface potential. Unlike the WT, large areas of positive charge are observed on the surface of the E219K. These variations of surface electrostatic potential between the two proteins are mostly clustered around the site of substitution, at the interface of β_2_-α_2_ loop and the C-terminal end of α_3_-helix, and at the N-terminal part of α_3_-helix. In the WT the area around the residue at position 219 is negatively charged or neutral, whereas in E219K the corresponding region is mostly positively charged.

In conclusion, the NMR structure of the E219K polymorphism suggests that the structural determinants for its dominant-negative effect are enciphered by the perturbation of the surface charge, and by subtle structural rearrangements most prominently localized in the β_2_-α_2_ loop region and at the interface of β_1_-α_1_ loop, α_1_-helix, α_1_-β_2_ loop and α_3_-helix. Variations in the distribution of electrostatic potential on the surface of E219K with respect to the WT are remarkable, considering that only individuals heterozygous at codon 219 seem resistant to sCJD. Our findings suggest that different distribution of charges in the WT and E219K may facilitate intermolecular interactions between the two allelic variants, thus sequestering them from the early stages of the fibrillization process or inhibiting interactions with yet unknown facilitators of prion conversion.

### 6.4. Common Structural Traits in HuPrP Pathological Mutants

The structural comparison of the mutants with the WT HuPrP structures allowed us to draw some preliminary conclusions towards the identification of common regions involved in the early stage of the conversion process to PrP^Sc^. The observed variations are mostly clustered at the interface between the β_2_–α_2_ loop and α_3_-helix. In particular, mutations alter the stabilizing interactions occurring in this region by promoting the progressive separation of the loop from the C-terminal part of α_3_-helix to the solvent. This increased distance upon mutation is clearly illustrated in Figure 3a,b. While in WT HuPrP solved at pH 5.5 the distance between Met166 and Tyr218 is 6.4 ± 0.1 Å, in Q212P and V210I it greatly increases up to 14.7 ± 0.4 Å. It is noteworthy that in E219K the same residues are much more closed, thus indicating that the protective polymorphism exhibits a more compact packing at the β_2_–α_2_ loop interface. Interestingly, the β_2_-α_2_ loop in E219K displays a tendency toward 3_10_-helical conformation, thus being somewhat better defined with respect to the same region in the WT and other disease-related mutants. These local structural features of the polymorphism may further account for its protective influence against sCJD.

Additionally, unlike the WT and E219K, the pathological mutants are characterized by exposure of hydrophobic and aromatic residues to the solvent in different structural regions. At acidic pH, in V210I the hydrophobic residues (Ile139, Met154 and Pro158) are much more solvent exposed at the α_1_-α_3_ interface rather than the same mutant structure at pH 7.2 (Figure 3c,d). Conversely, the Q212P showed increased exposition of the hydrophobic core in the β_2_-α_2_ loop and C-terminal part of α_3_-helix (Figure 3c,d).

Several groups investigated by molecular dynamics the structural effect of different HuPrP pathological mutants, including also V210I, E200K, Q212P and E219K [32,33,148,149,150]. These studies highlight the possible role of mutations on the flexibility of the β_2_–α_2_ loop and on the hydrophobic core organization, corroborating the NMR results.

## 7. Conclusions

Experimental evidence supports the hypothesis that the increased hydrophobicity of PrP^C^ is one of the main determinants of toxicity. Earlier studies proposed a mechanism of neurotoxicity through a direct interaction of PrP^Sc^, or partially unfolded intermediate states, with neuronal membranes [151,152], supporting the hypothesis that altered membrane association of PrP^C^ may be an important factor during the conversion to prion [153,154]. By means of spectroscopic and cellular techniques, several authors have shown that mild denaturation of recHuPrP leads to an increased exposure of the hydrophobic region that facilitates internalization and neurotoxic intracellular accumulation [155,156]. Cell treatment with recombinant HuPrP carrying disease-related mutations induces higher intracellular retention and neurotoxicity of both native and partially denaturated conformations [157]. Protein folding metastable intermediates are usually characterized by a significant exposure of hydrophobic residues which facilitates intermolecular interactions [158,159,160]. It has been proposed that the membrane environments may alter the PrP^C^ conformational structure. Spectroscopic studies performed on α-helix folded full-length recHuPrP showed a direct interaction of the protein with acidic vesicles containing acidic phosphatidylserine (POPS). The binding to POPS seems to occur at mildly acidic pH values and it involves only the N-terminal segment, while the globular domain retains its native conformation [161]. In further studies, the effect of different types of lipid membranes on recPrP structure was investigated by circular dichroism and Fourier transform infra red spectroscopy experiments [162,163]. These data showed that recPrP maintains its α-helix conformation when interacting with lipid raft-like membranes rich in cholesterol and sphingomyelin at pH 7. However, the binding of the protein to negatively charged lipid vesicles, such as the POPG membranes, promotes structural changes in β-sheet enriched structures at pH 5 and, to a lesser extent, at pH 7. Thus, the presented results clearly indicate that the preferred PrP^C^ localization in the lipid raft domains contributes to its stabilization in the α-helix structure. Altered PrP association with different lipid membranes, such as those in the endocytic compartments, may contribute to destabilize the native PrP^C^ fold.

In conclusion, our findings on disease-associated mutants and the E219K polymorphism provide new clues on the possible early events of HuPrP misfolding. The structural disorder of the β_2_–α_2_ loop, together with the increased distance between this loop and α_3_-helix observed in different mutants, lead to exposure of hydrophobic residues to solvent, thus facilitating intermolecular interactions with other PrP or with yet unknown cellular factors and abnormal accumulation inside the cells.
